# Beneficial effects of environmental enrichment and food entrainment in the R6/2 mouse model of Huntington's disease

**DOI:** 10.1002/brb3.235

**Published:** 2014-07-17

**Authors:** Elizabeth A Skillings, Nigel I Wood, A Jennifer Morton

**Affiliations:** Department of Physiology, Development and Neuroscience, University of CambridgeCambridge, CB2 3DY, UK

**Keywords:** Circadian restoration, FEO, metabolism, phenotype, voluntary exercise

## Abstract

**Background:**

In addition to their cognitive and motor deficits, R6/2 mice show a progressive disintegration in circadian rhythms that mirrors the problems associated with sleep-wake disturbances experienced by patients with Huntington's disease (HD). It has been shown previously that motor and cognitive performance, as well as survival, can be improved in transgenic mouse models of HD through the provision of environmental enrichment.

**Methods:**

We compared the effect of two different overnight entrainment paradigms presented either separately or in combination. The first was environmental enrichment, the second was temporal food-entrainment. Environmental enrichment was provided in the dark period (the natural active period for mice) in the form of access to a Perspex playground containing running wheels, tunnels, climbing frame, ropes and chew blocks. Food entrainment was imposed by allowing access to food only during the dark period. We assessed a number of different aspects of function in the mice, measuring general health (by SHIRPA testing, body temperature and body weight measurements), cognitive performance in the touchscreen and locomotor behavior in the open field.

**Results:**

There were no significant differences in cognitive performance between groups on different schedules. Environmental enrichment delayed the onset of general health deterioration, while food entrainment slowed the loss of body weight, aided the maintenance of body temperature and improved locomotor behavior. Effects were limited however, and in combination had deleterious effects on survival.

**Conclusions:**

Our results support previous studies showing that environmental enrichment can be beneficial and might be used to enhance the quality of life of HD patients. However, improvements are selective and ‘enrichment’ per se is likely to only be useful as an adjunct to a more direct therapy.

## Introduction

Huntington's disease (HD) is a neurodegenerative disorder caused by an expanded CAG repeat in the HD gene (The Huntington's Disease Collaborative Research Group 1993) that results in progressive striatal and cortical degeneration (Vonsattel and DiFiglia 1998; Reddy et al. 1999). Characteristic symptoms in HD encompass a range of progressive motor, cognitive, and psychiatric domains (for overview, see Bates et al. 2002). There is currently only one licensed treatment available for HD, the vesicular monoamine transport inhibitor tetrabenazine, that targets only chorea (Jankovic and Clarence-Smith 2011). In the absence of drug treatments other forms of therapy have been tested in patients. A form of enrichment known as remotivation therapy (a group therapy technique designed to motivate and engage nonverbal, withdrawn, and apathetic HD patients), has been shown to improve quality of life through increased self-esteem and self-awareness, and the restoration and maintenance of social functioning, even in late-stage HD (Sullivan et al. 2001). Khalil et al. (2013) have also shown a structured exercise program to be beneficial for patients with early to midstage HD, with improvements in gait speed, balance, and physical functioning. It has also been shown that leading a more active life (both physically and mentally) may protect against dementia and Alzheimer's disease (Fratiglioni et al. 2004; Verghese et al. 2003; Larson et al. 2006; Rovio et al. 2005), and delay the onset of HD (Trembath et al. 2010). Such lifestyle modifications can be simulated in laboratory animals through the provision of environmental enrichment (EE). Indeed, it has been demonstrated in a number of transgenic mouse models of HD that motor performance, cognitive performance, and survival can be improved through provision of EE (Carter et al. 2000; van Dellen et al. 2000; Hockly et al. 2002; Pang et al. 2006; Wood et al. 2010, 2011). In the majority of these studies, EE was provided through enhancement of basic housing. However, it has been suggested that provision of additional EE offering physical and cognitive stimulation may result in greater improvements. The concept of the cognitive reserve was first proposed by Stern (2002), and stemmed from the discovery that there often seemed to be little correlation between the levels of brain pathology and the actual clinical manifestation of damage caused by a disease. Subsequently, several authors (Jankowsky et al. 2005; Nithianantharajah and Hannan 2006; Mandolesi et al. 2008; Petrosini et al. 2009) have discussed the way in which prolonged exposure to EE, providing enhanced social, sensorimotor, and cognitive stimulation, is thought to cause changes in brain biochemistry, synaptic connectivity, and neuronal function that result in improved behavioral performance and maintenance. This was supported by the study of Wood et al. (2011) who found that cognitive enrichment in the form of a novel “noughts and crosses” maze not only increased survival in R6/2 mice but also improved cognitive performance when subsequently tested in a different task (Lashley III maze).

As well as cognitive and psychiatric symptoms, HD patients show a progressive disintegration of circadian rhythms (Goodman et al. 2010). These sleep–wake disturbances are mirrored in a number of lines of HD mice including R6/2, R6/1, Q175, and BAC HD (Morton et al. 2005; Kudo et al. 2011; Cuesta et al. 2012). Modulation of sleep–wake cycles through pharmacological imposition of sleep has been shown to improve cognitive function in R6/2 mice (Pallier et al. 2007; Pallier and Morton 2009). As peripheral clocks regulate gene expression cycles directly controlling physiological and metabolic rhythms (Kornmann et al. 2007), it is likely that the circadian disturbances seen in HD could have an effect on metabolism as well as behavior and that restoration of these cycles could also have beneficial consequences for physiological parameters such as body weight through improved metabolic efficiency. Maywood et al. (2010) have shown that imposing a temporally restricted feeding schedule, where food availability is limited to a specific time of day, can restore circadian behavioral and metabolic cycles in “late-stage” R6/2 mice through the activation of a food-entrainable oscillator (FEO). In the clinical setting, provision of palliative care for HD patients can offer broad scope for improvements in quality of life. For example, giving patients meals on a regular schedule, along with the provision of feeding assistance, might help stimulate the FEO in patients, improving circadian rhythms. Similarly, exercise is beneficial for both cardiovascular function and the maintenance and improvement of cognitive function, as long as it is not stressful or too strenuous for the patients.

In this study, we sought to investigate these effects further by exposing R6/2 mice to two different overnight schedules of entrainment, provided in the form of either environmental enrichment or temporal food entrainment, or a combination of the two.

## Material and Methods

### Animals

All experiments were conducted in accordance with the United Kingdom Animals (Scientific Procedures) Act, 1986. Lighting was controlled on a 10 h light:14 h dark cycle corresponding to a short daylength photoperiod, with the light period starting at 08:00 h. This allowed the other mice to be placed into and removed from the playgrounds and the point of lights off/on. All behavioral testing was conducted during the light phase. The housing facility temperature was maintained at 21–23°C and the relative humidity was controlled (55 ± 10%). Forty-nine mice (details given below) were taken from colonies of R6/2 mice established in the University of Cambridge and maintained by backcrossing onto CBA × C57BL6 F1 female mice. The mice had CAG repeats of 250 ± 17 (mean ± SEM). Genotyping was performed by PCR from tail snips taken at 3 weeks of age and CAG repeat lengths were measured by Laragen (Los Angeles, CA). All CAG repeat numbers reported are those determined directly by Genemapper software (Life Technologies, Grand Island, NY). Mice had ad libitum access to water via lowered waterspouts and standard dry laboratory food. Our mice live as standard in an enriched environment with increased amounts of bedding and nesting materials. In addition, mice were given a supplementary feed of “mash” (pellets soaked in water overnight) twice daily, unless otherwise stated.

### Microchip implantation and temperature recording

At 7 weeks of age, implantable electronic transponders (IPTT300, Bio Medic Data Systems Inc., Seaford, DE) were injected subcutaneously into the mice. These were used for identification and to record body temperature by scanning the animal using the IPTT6005 scanner probe (Bio Medic Data Systems Inc.). From 8 weeks of age until death, body temperatures were recorded twice daily, corresponding to the start of light and dark periods at 08:00 (lights on) and 18:00 (lights off). Weekly averages for each time point were calculated for each animal. Eight-week body temperatures of R6/2 mice taken prior to commencement of treatment were pooled for analysis. Data are plotted as blocks of 7 days from 17 to 22 weeks of age.

### Experimental groups

Groups consisted of 9 or 10 age-matched male mice housed in single-genotype cages. Mice were split into five different groups. These were a group of WT and a group of R6/2 mice that were used as controls (no enrichment and ad libitum access to food), and three groups of R6/2 entrained animals as follows:

1. Control WT—ad libitum food and no environmental enrichment (WT CTRL).
2. Control R6/2—ad libitum food and no environmental enrichment (R6/2 CTRL).
3. R6/2—ad libitum food with environmental enrichment (R6/2 EE).
4. R6/2—food-entrainment schedule but no environmental enrichment (R6/2 FE).
5. R6/2—food-entrainment schedule plus environmental enrichment (R6/2 EE:FE).

### Food entrainment

Mice on a food-entrainment schedule received ad libitum access to dry food pellets and approximately 2 g of “mash” per mouse from 18:00 to 08:00. Pellets and mash were placed in the cage or playground at the start of the dark period and any uneaten food was removed at the start of the following light period. Control mice that were not food entrained received dry food pellets ad libitum and were given mash (2 g per mouse) twice daily at approximately 09:00 and 15:00.

### Environmental enrichment

**Figure 1:**
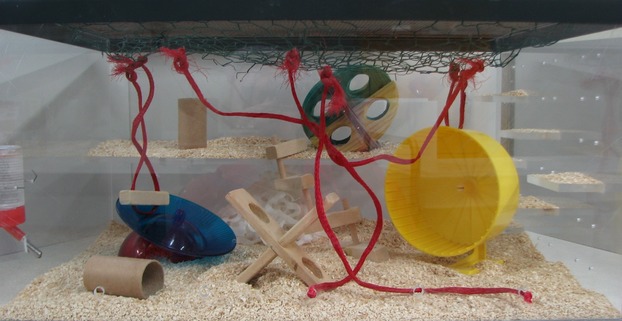
Environmental enrichment setup. Playground contained a combination of ropes, ladders, running wheels and wooden toys. The combination and configuration of toys was changed daily to maximise stimulation.

Environmental enrichment was provided in the form of overnight (18:00 h to 08:00 h) access to Perspex™ playgrounds (60 × 30 × 45 cm) containing a combination of ropes, ladders, running wheels, and toys (Fig. 1). The combination of toys and their configuration were changed each day to maximize the stimulating nature of the environment. Between sessions, toys were cleaned with a solution of 1% acetic acid.

### SHIRPA

We used a modified version of the phenotype assessment (SHIRPA) protocol (SmithKline Beecham Pharmaceuticals; Harwell, MRC Mouse Genome Center and Mammalian Genetics Unit; Imperial College School of Medicine at St Mary's; Royal London Hospital, St Bartholomew's and the Royal London School of Medicine). The protocol used for SHIRPA testing is outlined in Glynn et al. (2003). Mice were tested at 8 weeks (prior to entrainment), 12 weeks (prior to cognitive testing), 16 weeks (post cognitive testing), and finally at 20 weeks of age. Data were quantified using a binary scoring system where a “normal” behavior received a score of 0 and an “abnormal” behavior received a score of 1. A cumulative score was then determined for each mouse. Higher scores indicate a larger degree of abnormality.

### Open field

Mice were evaluated in the open-field task as described by Carter et al. (2000). Briefly, individual mice were placed in an open-topped plywood box 60 × 60 × 30 cm high, with a white floor marked with black gridlines (divided into 25 squares, each measuring 12 × 12 cm). Individual mice were placed in the central square of the open field and observed for a 15-min period. Parameters measured within the 10-min period include: (1) latency to reach the periphery; (2) total number of central squares entered (defined as three or more paws moving into a central square); (3) total number of peripheral squares entered (defined as three or more paws moving into a peripheral square); (4) total number of squares entered; (5) total incidences of supported rearing (standing up on hind legs using the wall for support); (6) total incidences of unsupported rearing (standing up on hind legs without wall support); (7) number of complete grooming cycles; (8) number of fecal boli; and (9) number of urinations.

### Touch-screen testing

Preliminary training and behavioral testing were carried out in 12 automated touch-screen testing chambers. The apparatus consisted of an infrared touch screen (Craft Data Ltd., Bucks, UK) and a standard modular testing chamber housed within a sound-attenuating box (Med Associates Inc., St Albans, VT). The box was fitted with a 28 volt DC fan for ventilation and masking of extraneous noise. The inner operant chamber (21.6 × 17.8 × 12.7 cm; Med Associates Inc.) consisted of a metal frame, clear Perspex walls, and a stainless steel grid floor. A pellet receptacle (magazine) attached to a 14 mg pellet dispenser was situated outside of the box. A 3 W houselight and tone generator (Med Associates Inc.) were fitted to the back wall of the chamber. The magazine was illuminated by a 3 W light bulb and fitted with photocell head entry detectors to detect the presence of a mouse in that area of the testing chamber. Stimuli were presented on an infrared touch screen located at the opposite end of the chamber. A Perspex “mask” containing two response windows in which the stimuli were displayed was positioned in front of the touch screen, approximately 1.6 cm from the floor of the chamber, to stop the mouse from accidentally triggering the touch screen with its tail. From 3 days prior to, and throughout the 4 weeks of cognitive testing, mice were placed on a restricted diet, but maintained such that their body weight was no less than 85% of the free-feeding body weight of a standard R6/2 mouse. Mice were fed mash approximately 1 h post testing for free-feeding mice and overnight for food-restricted mice. We used a similar task for behavioral testing as described previously (Morton et al. 2006). Briefly, mice began discrimination training at 12 weeks of age and were given 14 daily sessions of 30 two-choice discrimination trials after which the stimuli were reversed and mice were given a further 14 daily sessions of 30 two-choice discrimination trials with this new stimulus-reward contingency.

### Body weights and survival

Body weights were recorded daily from 8 weeks of age until the last R6/2 mouse had died or was killed for humane reasons. Although data were collected until end stage, here data are only presented up to 22 weeks, since after this time a significant number of mice had died or been killed and could not be included in longitudinal analysis. The period when cognitive testing was taking place and all mice received a restricted diet is represented in Figure 4 by a shaded area. Age of death was recorded for all R6/2 mice. Mice were killed when they reached end point—if they were moribund, lacked a righting reflex, exhibited signs of pain (abnormal vocalization), or failed to respond to gentle stimulation.

### Statistical analysis

Statistical analyses were performed using Statsoft Statistica v11 software (Statsoft, Tulsa, OK) or Prism 5 (GraphPad Software Inc., San Diego, CA). At the 8-weeks time point, prior to commencement of treatment, all R6/2 groups were pooled for analysis. Subsequently, only the R6/2 CTRL group was analyzed in direct comparison to the WT CTRL group. For statistical analysis of cognitive testing, body weights, and temperatures, we used repeated measures ANOVA (factors: enriched/not enriched and food entrained/not food entrained). SHIRPA abnormality scores were analyzed using a Wilcoxon matched pair test. Open-field data were analyzed using a univariate analysis (factors: enriched/not enriched and food entrained/not food entrained for R6/2 comparisons and genotype for WT/R6/2 CTRL comparisons), and survival data using a log-rank test. Bonferroni's post hoc test was used to determine specific differences, when significant group effects were found.

Significance levels were set at *P* < 0.05 for all analyses.

## Results

### Mice on a food-entrainment schedule show increased locomotion and exploration behaviors in the open field

**Figure 2:**
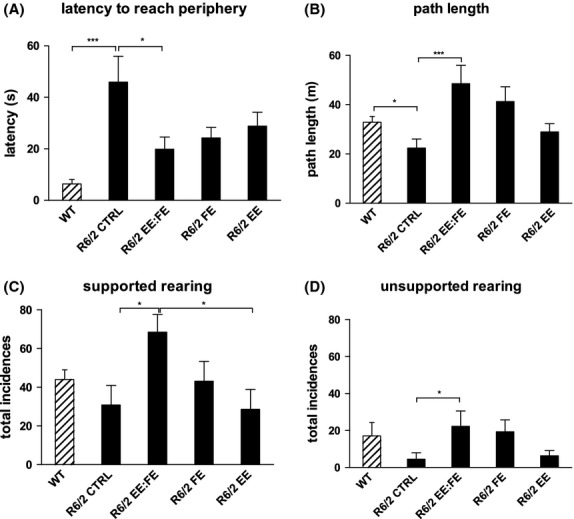
Open field performance of mice aged 19 weeks. Results shown are latency to periphery (A), path length (B), supported rearing (C) and unsupported rearing (D). EE:FE, enriched:food entrained; FE, food entrained; EE, enriched. Bars are means ± SEM. *P < 0.05, **P < 0.01, ***P < 0.001.

When first placed in the open field, WT mice move quickly to the periphery (Fig. 2A) and displayed investigative behaviors, such as supported (front paws resting on the walls of the open field) and unsupported rearing (Fig. 2C and D). WT CTRL were quicker than R6/2 CTRL mice to move from the centre to the periphery (Fig. 2A, *F*_1,18_ = 16.67864, *P* < 0.001). R6/2 CTRL mice also showed reduced levels of locomotor activity, as measured by path length (m) (Fig. 2B, *F*_1,18_ = 6.6247, *P* < 0.05) compared to WT CTRL mice.

We also found a number of differences between R6/2 CTRL mice and those on the combined food-entrainment and environmental schedule. R6/2 EE:FE mice were faster to move to the periphery (*F*_1,33_ = 5.7106, *P* < 0.05; Fig. 2B) and had an increased path length (*F*_1,33_ = 13.304, *P* < 0.001) compared to R6/2 CTRL mice. Further improvements in locomotor behavior in the R6/2 EE:FE group were demonstrated by increased exploratory behavior, shown as a greater incidence of supported rearing behavior compared to both R6/2 CTRL and R6/2 EE mice (*F*_1,33_ = 7.5656, *P* < 0.01; Fig. 2C), and unsupported rearing compared to R6/2 CTRL mice (*F*_1,33_ = 7.2472, *P* < 0.05; Fig. 2D).

### Environmental enrichment and food entrainment delay phenotype progression

**Figure 3:**
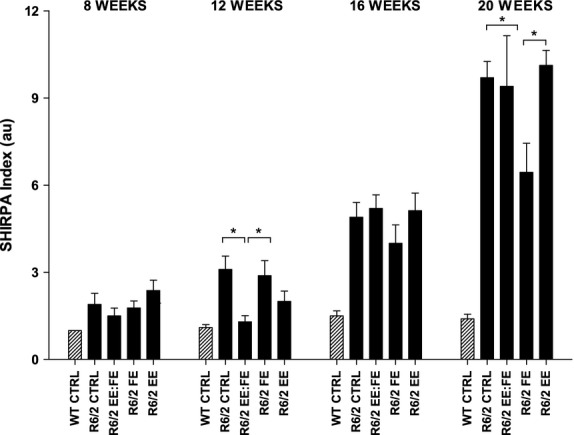
SHIRPA scores. SHIRPA abnormality indices from testing conducted at 8, 12, 16 and 20 weeks of age. Error bars are not present for WT CTRL mice at 8 weeks of age as all mice received a SHIRPA score of 1. Units are absolute values (a.u.) based on SHIRPA scoring and are not scaled to WT levels. EE:FE, environmental enrichment:food entrained; FE, food entrained only; EE, environmental enrichment only. Bars are means ± SEM. *P < 0.05.

WT control mice typically had a mean SHIRPA index of 1 at all time points (Fig. 3), something which is quite usual at this point, reflecting their normal phenotype (smooth coat, standard gait, etc.). While there was no significant difference in the SHIRPA index of any of the groups up to 8 weeks of age (prior to treatment), after this point the phenotype of the R6/2 CTRL mice began to deteriorate, as reflected by their higher SHIRPA index. At 12 weeks of age, a beneficial effect on SHIRPA index was seen in R6/2 EE:FE mice compared to both the R6/2 CTRL (*z* = 2.42, *P* < 0.05) and the R6/2 FE mice (*z* = 2.02, *P* < 0.05; Fig. 3). This effect had disappeared by 16 weeks of age. At 20 weeks of age, however, R6/2 FE mice had a significantly lower abnormality index than R6/2 CTRL (*z* = 2.52, *P* < 0.05) and R6/2 EE groups (*z* = 2.38, *P* < 0.05; Fig. 3).

### Mice receiving environmental enrichment have lower body weights

**Figure 4:**
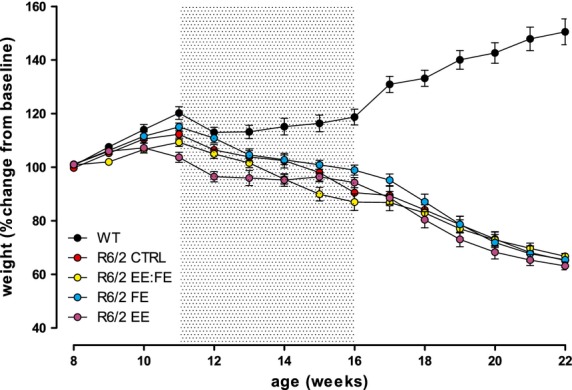
Body weights. Only data up to 22 weeks is shown as deaths of mice after this point made analysis problematic. Data are plotted in blocks of 2 weeks. Shaded area represents the period when cognitive testing was taking place and all mice received a restricted diet. Data are means ± SEM. Where error bars are not visible, they are obscured by symbols. EE:FE, environmental enrichment:food entrained; FE, food entrained only; EE, environmental enrichment only.

As expected, due to the effect of the mutation, all R6/2 mice had lower body weights than their WT counterparts (*F*_1,17_ = 59.381, *P* < 0.001; Fig. 4). In addition, all groups of mice showed a general decrease in body weight from 11 to 12 weeks of age as a result of initial food restriction required for cognitive testing. However, once cognitive testing had been completed and mice returned to a standard feeding regime, body weights of WT mice steadily increased, while those of mice in R6/2 groups continued to decline. There was a negative effect of environmental enrichment throughout the experiment, with R6/2 EE and R6/2 EE:FE mice having significantly lower body weights (as a percentage of baseline) than all other groups (*F*_1,19_ = 5.518, *P* < 0.05; Fig. 4).

### Food entrainment aids maintenance of body temperatures at older ages

#### Daily body temperatures

**Figure 5:**
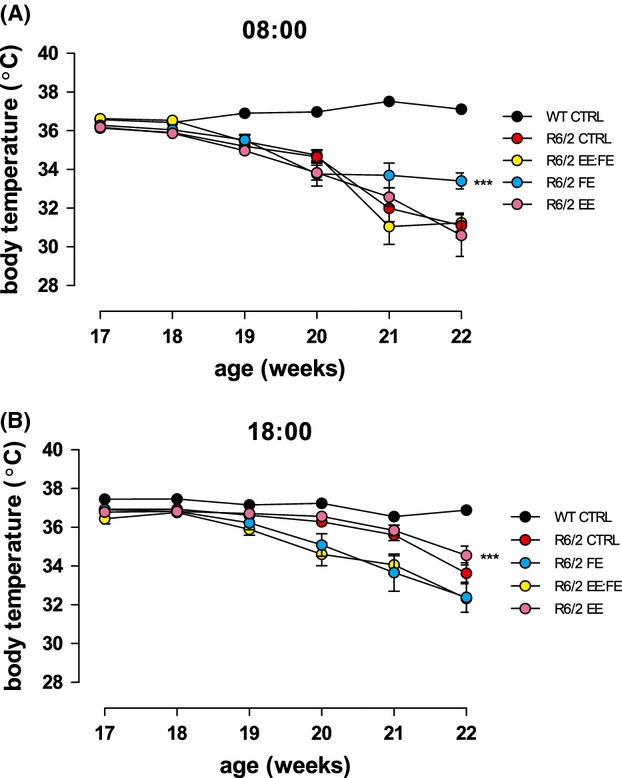
Daily body temperatures. Temperatures were measured at 08:00 (A) and 18:00 (B). Data are plotted as blocks of 7 days from 17 to 22 weeks of age. Where error bars are not visible, they are obscured by symbols. EE:FE, enriched:food entrained; FE, food entrained; EE, enriched. Data are means ± SEM. ***P < 0.001.

Mean daily body temperatures of mice aged 17–22 weeks are shown in Figure 5. Throughout the study the body temperatures of WT CTRL mice were consistently higher than that of the R6/2 CTRL mice at both 08:00 (*F*_1,15_ = 69.990, *P* < 0.001) and 18:00 (*F*_1,15_ = 10.460, *P* < 0.01). At 22 weeks of age, differences in the body temperatures of R6/2 groups began to emerge at both 08:00 (*F*_42,224_ = 2.4673, *P* < 0.001) and 18:00 measurements (*F*_42,224_ = 2.6819, *P* < 0.001). In the measurements taken at 08:00, R6/2 FE mice had higher body temperatures than both R6/2 CTRL (*P* < 0.05) and R6/2 EE (*P* < 0.001). In the temperatures recorded at 18:00, there was the opposite effect. R6/2 EE mice had significantly higher body temperatures than both R6/2 EE:FE (*P* < 0.01) and R6/2 FE (*P* < 0.001) at 22 weeks of age.

### Neither environmental enrichment nor food entrainment improved cognitive function

**Figure 6:**
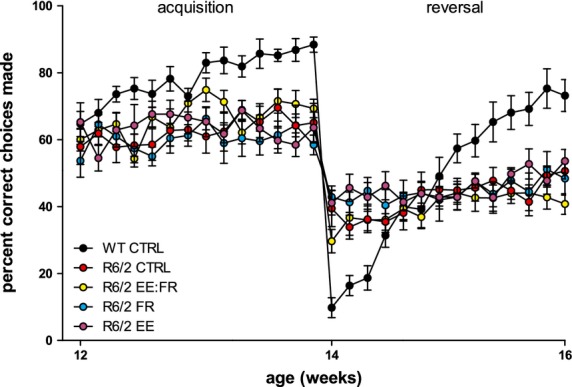
Performance of mice tested on the acquisition and subsequent reversal of a two-choice discrimination task in the touchscreen. Where error bars are not visible, they are obscured by symbols. EE:FE, enriched:food entrained; FE, food entrained; EE, enriched. Data are means ± SEM.

Environmental enrichment has been shown to improve cognitive ability in previous studies using R6/2 mice. In order to assess this, mice were tested in the touch screen from 12 weeks of age, however, while all WT mice successfully completed the task, none of the R6/2 groups were able to learn either acquisition or reversal (Fig. 6).

### An environmental enrichment schedule combined with food entrainment reduces survival

**Figure 7:**
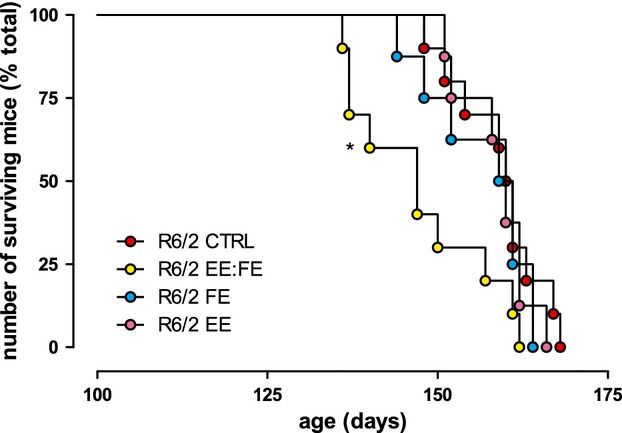
Survival of mice. EE:FE, enriched:food entrained; FE, food en-trained; EE, enriched. *P < 0.05.

No WT mice died during the course of the study. All R6/2 mice had died by 24 weeks of age. However, R6/2 EE:FE mice died significantly earlier than all other transgenic groups (χ^2^ = 11.86, *P* < 0.001; Fig. 7).

## Discussion

It has been shown that an active lifestyle (involving a combination of social, physical, and cognitive components) protects against dementia and Alzheimer's disease in human patients (Fratiglioni et al. 2004; Rovio et al. 2005). Although several theories have been proposed to explain the beneficial effects of EE, the exact mechanism remains unclear. One suggestion put forward is that EE is able to rescue entirely the downregulation of hippocampal and striatal levels of brain-derived neurotrophic factor (BDNF) that is seen in HD (Spires et al. 2004; Angelucci et al. 2009). Other proposed benefits of EE include enhanced hippocampal neurogenesis (van Praag et al. 1999; Brown et al. 2003; Lazic et al. 2006), decreased intranuclear inclusion load (Benn et al. 2010) and positive effects on neurotransmitters including increased levels of dopamine release (Segovia et al. 2008a) and reduced levels of acetylcholine in the prefrontal cortex (Segovia et al. 2008b) and increased GABA and glutamate levels in the hippocampus (Segovia et al. 2006). Recent studies from our laboratory have shown that there are beneficial effects of environmental enrichment on cognitive performance, body weights, and survival (Wood et al. 2010) and that treatments such as food-entrainment paradigms, voluntary exercise regimes, and bright light therapy can restore circadian rhythms (Maywood et al. 2010; Cuesta et al. 2014) in R6/2 mice. Here, we sought to investigate the beneficial effects of these treatments further by administering a combination of environmental enrichment and food-entrainment paradigms.

In this study, mice on a food-entrainment schedule showed an improvement in phenotype as shown by improved SHIRPA scores (R6/2 EE:FE at 12 weeks and R6/2 FE at 20 weeks of age), in addition to increased locomotion and exploration behaviors found in the open field (R6/2 EE:FE mice only). R6/2 mice show progressive atrophy of skeletal muscle, particularly in the later stages of the disease, with mice developing cachexia, similar to that seen in late-stage HD patients (Ribchester et al. 2004). Maywood et al. (2010) suggest that as skeletal muscle has one of the highest metabolic demands (after the brain and heart), the deterioration of metabolic circadian rhythms in R6/2 mice could significantly contribute to this aspect of the HD phenotype. They demonstrated that by enforced scheduling of feeding behavior, the action of the food-entrainable oscillator (FEO) could be exploited in order to restore behavioral and metabolic circadian rhythms. We propose that it is by restoration of the circadian rhythms, through a fixed-period feeding schedule, that the delay in phenotype development and deterioration of locomotor function was seen in our food-entrained mice. This is in line with the findings of Duan et al. (2003), who showed that huntingtin-mutant mice maintained on a restricted diet were better able to maintain body weight than those fed ad libitum. They suggested that dietary restriction is able to suppress the pathological processes which underlie muscle atrophy. Positive effects of environmental enrichment have been found in WT mice tested in the open field, with improvements in locomotor and exploratory behaviors (Tsai et al. 2003; Nithianantharajah et al. 2008). It is possible that the lack of improvement in the open field seen in R6/2 EE mice was due to testing taking place during their usual sleep period, which may have led to a degree of sleep deprivation. It may be that mice which had spent an active night in a more stimulating environment such as the playground were more tired during the light period (when nocturnal animals typically sleep). Testing was conducted during this period and open-field performance may have been worse as a result. Given that open-field performance was improved in R6/2 EE:FE mice which received environmental enrichment in combination with a food-entrainment regime, it seems more likely that improvements were a direct result of circadian restoration provided by food entrainment rather than any beneficial consequences of EE.

It is thought that weight loss in the R6/2 mouse is the result of an increased metabolism (van der Burg et al. 2008). Food entrainment for this study required mice in EE:FE and FE groups to be temporally food restricted, something which could have ultimately proved to be detrimental to body weights. However, there is no evidence that this was the case. While all mice in this study had lower body weights from 12 to 16 weeks during cognitive testing as a result of the food restriction required for touch-screen testing, body weight loss (% change from baseline) in R6/2 FE mice was not significantly greater than that of any other R6/2 group. Throughout the study it was those mice receiving environmental enrichment which had lower body weights compared to all other mice. Body weights of WT mice have been shown previously to be unaffected by environmental enrichment (Tsai et al. 2003; Wood et al. 2010). Given the long time frame over which enrichment took place this reduced body weight may be simply due to the increased metabolism seen in R6/2 mice in combination with the higher exercise levels from EE as seen previously (Wood et al. 2011).

We have shown recently that environmental enrichment can have both beneficial (Wood et al. 2010) and detrimental (Wood et al. 2011) effects on survival. In the current experiment, while R6/2 EE:FE mice showed a delayed onset of phenotype as indicated by lower SHIRPA scores at 12 weeks of age, ultimately this did not translate to an increased survival rate and these mice died significantly earlier than all other R6/2 groups. R6/2 mice in the EE:FE group died of their disease in the same manner as all other R6/2 mice in the study, with no noted incidences of aggression or fighting. However, the lower body weights of enriched mice throughout the study, combined with the lower body temperatures caused by food entrainment from 20 weeks of age may be a causal factor. This may have left them less able to manage the stress effects resulting from increased competition for the greater number of resources available within the playground environment compared to the home cage. While we perceive an environment filled with toys to be enriching, a mouse may feel an increased need to protect its new environment from cage mates, increasing levels of stress and aggression, something which is known to induce weight loss in rodents (Harris et al. 2002). The daily change in home environment from cage to playground may in itself induce stress effects. Gerdin et al. (2012) investigated the impact of stress resulting from common husbandry and experimental procedures on mouse welfare, looking at parameters such as systolic arterial pressure, heart rate, and locomotor activity. They found that mice do not habituate to basic changes to the cage environment regardless of the frequency with which changes take place. Given that the provision of environmental enrichment is widely reported to ameliorate stress, anxiety, and depressive-like behaviors (Morley-Fletcher et al. 2003; Ilin and Richter-Levin 2009; Pang et al. 2009; Jha et al. 2011; Du et al. 2012; Renoir et al. 2012, 2013), it may be useful in future experiments to measure corticosterone levels to test this hypothesis or to provide enrichment within the home cage environment itself, to reduce the potential for stress effects.

In this study, mice began to have significant drops in body temperature from around 20 weeks of age. R6/2 mice are known to have deficits in thermoregulation associated with impaired activation of brown adipose tissue and show a progressive drop in body temperatures as the disease manifests itself (Weydt et al. 2006). We know that body temperature fluctuates according to the time of day driven by a circadian pacemaker and can be used as a marker of circadian synchronization (Kelly 2006). We found that the body temperatures of R6/2 mice were affected to varying degrees by both environmental enrichment and food entrainment. In the daily temperature recordings taken at 08:00, R6/2 FE mice had higher body temperatures compared to CTRL and R6/2 EE mice at 22 weeks of age. It is possible that the increased metabolism seen in R6/2 mice (van der Burg et al. 2008) causes the lower body temperatures, maybe as a result of lower body weights. Exploitation of the FEO through the food-entrainment schedule may have resulted in a delay in this dysfunction, stabilizing the drop in body temperatures in these mice.

Cognitive testing was carried out using a two choice discrimination in the touch screen. We used this method as it has less motoric demand on the mouse than more traditional tasks (e.g., the two-choice swim tank) and we were concerned that a more strenuous task, and the resulting fatigue may have affected the ability of mice housed in the playgrounds to make full and active use of their environment. Neither environmental enrichment nor food entrainment improved cognitive performance. It is possible that this was due to the late age at which mice began testing (12 weeks), as previous studies (Morton et al. 2006) demonstrated that at this age mice are already showing impairments in reversal learning in the touch screen. As treatment did not begin until 8 weeks of age it was not possible to start cognitive testing earlier than this. It is probably easier to delay the onset of cognitive deficits than to try to restore them once they are established. As such, it may be beneficial to begin treatment at earlier ages in order to carry out testing earlier and to realize any beneficial effects on cognition. Alternatively, the use of a different task such as the two-choice swim tank, in which cognitive deficits appear later, may reveal beneficial effects.

The scale of improvements generated by environmental enrichment and food entrainment observed in this study may have been reduced by the fact that our mice are already housed in what many laboratories would refer to as enriched. Our mice are routinely provided with plastic housing, paper wool bedding, and lowered water spouts and their regular diet is also supplemented with a mash fed twice daily to improve hydration and enable mice with an advanced phenotype to gain access to sufficient amounts of food. Living in an enriched environment raises the baseline for improvements as it may have already produced some improvements in the phenotype of the mouse. Taking these factors into account, any phenotypic improvements need to be greatly increased before statistically significant improvements are seen, though this is not necessarily a confound, as starting with high baseline levels of enrichment makes our study more relevant to human patients where treatments yield positive results.

## Conclusion

The precise mechanism by which environmental enrichment and food entrainment affect disease progression in R6/2 mice remains unclear. While treatments yielded no improvements in cognition, environmental enrichment and food entrainment delayed the onset of phenotype, improved maintenance of body weight and body temperature, and improved locomotor behavior in the open field dependent on the combination in which they were administered. Effects were limited, however, and when administered as combination treatments, they had deleterious effects on survival. While increased environmental stimulation and maintenance of daily behavioral schedules could potentially be used therapeutically, improvements in short-term well-being are not necessarily beneficial in the longer term. This raises the possibility that there is a tradeoff between improvements in the quality rather than quantity of life, something which the medical profession and HD patients may view wholly differently.
